# Female Patients With Sleep-Disordered Breathing Display More Frequently Heart Failure With Preserved Ejection Fraction

**DOI:** 10.3389/fmed.2021.675987

**Published:** 2021-05-28

**Authors:** Simon Lebek, Philipp Hegner, Maria Tafelmeier, Leopold Rupprecht, Christof Schmid, Lars Siegfried Maier, Michael Arzt, Stefan Wagner

**Affiliations:** ^1^Department of Internal Medicine II, University Hospital Regensburg, Regensburg, Germany; ^2^Department of Cardiothoracic Surgery, University Hospital Regensburg, Regensburg, Germany

**Keywords:** sleep-disordered breathing, sex, gender, intermittent hypoxia, HFpEF, ACE2

## Abstract

**Objective:** Sleep-disordered breathing (SDB) is a widespread disease that is often associated with heart failure (HF) with preserved ejection fraction (HFpEF). HFpEF is more frequent in women than in men, but detailed pathomechanisms remain unclear. We investigated HFpEF in women and men in a high-risk cohort with SDB monitoring.

**Methods and Results:** Three hundred twenty-seven patients (84.4% men) undergoing elective coronary artery bypass grafting were prospectively subjected to SDB monitoring, and an apnea–hypopnea index (AHI) ≥15/h defined SDB. HF was classified according to current guidelines. HFpEF was significantly more frequent in SDB patients compared to those without SDB (28 vs. 17%, *P* = 0.016). This distribution was driven by an increased frequency of HFpEF in female SDB patients (48% vs. only 25% in male, *P* = 0.022). In accordance, female patients with SDB exhibited significantly more impaired diastolic left ventricular filling compared to men (echocardiographic E/e′). In contrast to men, in women, minimum oxygen saturation (O_2min_, measured by polygraphy, *R*^2^ = 0.470, *P* < 0.001) and time of oxygen saturation <90% (*R*^2^ = 0.165, *P* = 0.044) were significantly correlated with E/e′. Moreover, the correlation between O_2min_ and E/e′ was significantly different in women compared to men (*P* < 0.001). Intriguingly, this association remained independent of clinical covariates in women [age, body mass index, systolic contractile dysfunction, diabetes mellitus, and glomerular filtration rate (GFR), *R*^2^ = 0.534, *P* = 0.042, multivariate regression analysis]. Since angiotensin II signaling has been mechanistically linked to HF, we measured protein expression of its cleavage enzyme ACE2 in human right atrial appendage biopsies (Western blot). Intriguingly, we found a significantly decreased ACE2 expression preferentially in women with SDB (2.66 ± 0.42 vs. 4.01 ± 2.47 in men with SDB, P = 0.005). In accordance, left ventricular mass index was significantly increased in women with SDB compared to women without SDB.

**Conclusion:** In patients with SDB, HFpEF and diastolic dysfunction were more frequent in women compared to men. In contrast to men, the severity of SDB was associated with the degree of diastolic dysfunction in women. These insights might help to find sex-specific therapies for patients with sleep-disordered breathing and heart failure.

**Clinical Trial Registration:** Unique identifier: NCT02877745, URL: http://www.clinicaltrials.gov.

## Introduction

Sleep-disordered breathing (SDB) is a widespread disease with increasing prevalence and emerging socioeconomic relevance (1). The major drivers of the increasing prevalence are the aging population and the ongoing obesity epidemic (2). Unfortunately, SDB can contribute to hypertension (3) and is often associated with severe comorbidities that substantially worsen prognosis, like atrial fibrillation (4) or heart failure (HF) (5, 6). Both SDB and HF are especially frequent in high-risk patients, e.g., in patients with acute myocardial infarction (7) or in patients undergoing coronary artery bypass grafting (CABG) (5–8). In particular, HF-dependent congestion in the pulmonary circuit with subsequent pulmonary hypertension may also contribute to increased SDB severity (9).

HF can be classified into HF with reduced ejection fraction (HFrEF), mid-range ejection fraction, and preserved ejection fraction (HFpEF) (6). Interestingly, HFpEF is more frequent in women, and HFrEF is more likely to be found in men, while detailed mechanisms remain elusive (6, 10, 11). In addition, there is only sparse data regarding this important issue in high-risk patients with SDB. Therefore, there is an urgent need for further insights regarding SDB and HF in high-risk patients that may guide us to future therapeutic strategies. HFpEF has already been shown to be associated with left atrial enlargement and left ventricular hypertrophy (10, 11). Interestingly, the latter has been mechanistically linked to an increased angiotensin II signaling that can be alleviated by angiotensin II cleavage enzyme angiotensin-converting enzyme 2 (ACE2) (12).

The present study aims to characterize HFpEF and diastolic dysfunction in women and men with SDB of a high-risk patients' cohort undergoing CABG. Moreover, we measured ACE2 protein expression in a human myocardium as a potential sex-dependent pathway that may lead to hypertrophy and subsequent HFpEF.

## Materials and Methods

This is a cross-sectional experimental and clinical sub-study of the prospective observational study “Impact of sleep-disordered breathing on atrial fibrillation and perioperative complications in patients undergoing coronary artery bypass grafting surgery—a prospective observational study” (CONSIDER-AF: NCT02877745, https://clinicaltrials.gov/ct2/show/NCT02877745?term=NCT02877745&rank=1) (13).

### Study Approval and Design

**Figure 1:**
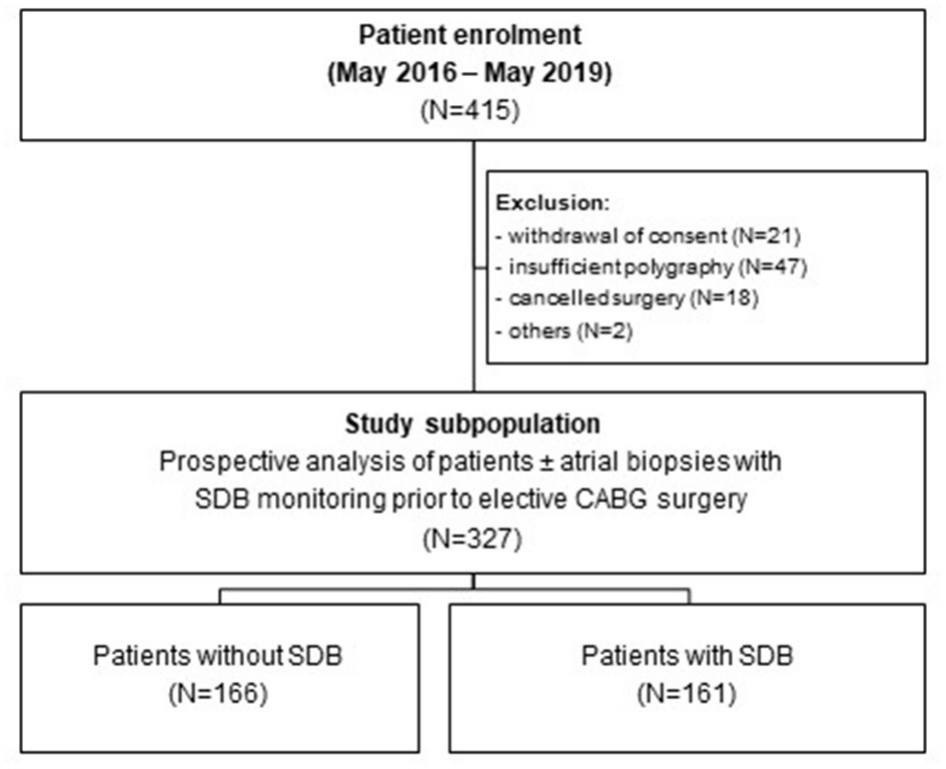
Study flowchart. Study flowchart showing the enrolment of 415 patients undergoing elective coronary artery bypass grafting. After exclusion of 88 patients, 166 patients without SDB and 161 patients with SDB (± right atrial appendage biopsies) were prospectively analyzed.

This study was approved by the local ethics committee (University of Regensburg, Bavaria, Germany) and is in accordance with the Declaration of Helsinki (first released in 1964, most recent revision 2013). Between May 2016 and May 2019, each patient that was scheduled for elective CABG at the University Hospital Regensburg was prospectively screened for eligibility. Age between 18 and 85 years, planned elective CABG, and provided written informed consent were inclusion criteria. Pre-specified exclusion criteria were preexisting treated SDB, nocturnal positive airway pressure support or mechanical ventilation, severe obstructive pulmonary disease, oxygen therapy, and preoperative use of inotropes or intra-aortic balloon pump. This resulted in 415 patients that were tested as eligible (Figure 1). Twenty-one patients withdrew their consent, 47 polygraphies were insufficient, 18 surgeries were canceled, and 2 other patients had to be excluded, resulting in a final study subpopulation of 327 patients from which echocardiographic data were available. Some patients (*N* = 99) donated a right atrial appendage biopsy for further experimental analyses. For optimal cardioplegia, biopsies were transported to our laboratories in ice-cold Custodiol® solution (with 2 mmol/L butanedione monoxime).

### Assessment of SDB

SDB was assessed using standard polygraphy during the night before surgery (Alice NightOne Device; Philips Respironics, Murrysville, USA). Airflow, blood oxygen levels, and respiratory efforts were measured. The acquired sleep data were analyzed by experienced medical staff using Sleepware G3 sleep diagnostic software (Philips Respironics, Murrysville, USA). As described previously, there was a ≥90% decrease in airflow for ≥10 s defined apneas (8). Hypopneas were defined as ≥30–90% decrease in airflow vs. baseline for ≥10 s and desaturation as a ≥4% decrease in oxygen saturation (14, 15). An apnea–hypopnea index (AHI, mean occurrence of apneas and hypopneas/h sleep) ≥15/h defined SDB. Mean number of desaturations/h sleep defined the oxygen desaturation index (8).

### Classification of HF

HF was classified according to current guidelines (6). HFrEF was defined by having symptoms and a left ventricular ejection fraction (LVEF) <40%. HF with mid-range ejection fraction was defined by having symptoms with LVEF 40–49% and elevated N-terminal-pro brain natriuretic peptide (NT-pro BNP) levels and either left ventricular hypertrophy (left ventricular mass index ≥115 g/m^2^ for male, ≥95 g/m^2^ for female patients), left atrial enlargement (left atrial volume index >34 ml/m^2^), or diastolic dysfunction. HFpEF was defined by having symptoms with LVEF ≥50% and elevated NT-pro BNP levels and either left ventricular hypertrophy, left atrial enlargement, or diastolic dysfunction.

### Western Blots

Human right atrial appendage biopsies were homogenized using Tris buffer, containing (mmol/L) 20 Tris–HCl, 200 NaCl, 20 NaF, 8.9 Nonidet P-40 (Sigma Aldrich), 18.3 phenylmethanesulfonylfluoride (Sigma Aldrich), complete protease inhibitor cocktail (Roche), and complete phosphatase inhibitor cocktail (Roche). Protein concentration was measured by a bicinchoninic acid (BCA) assay (Pierce Biotechnology). After denaturation [30 min at 37°C at 500 rpm in 2% β-mercaptoethanol (Sigma Aldrich)], proteins were separated on 8% sodium dodecyl sulfate (SDS)–polyacrylamide gels and transferred to a nitrocellulose membrane (GE Healthcare). Primary antibodies [rabbit monoclonal anti-ACE2 (1:5000, Abcam, catalog number ab108252) and mouse monoclonal antiglyceraldehyde 3-phosphate dehydrogenase (anti-GAPDH) (1:10,000, Sigma Aldrich, catalog number G8795)] were incubated at 4°C overnight. Secondary antibodies [horseradish peroxidase (HRP)-conjugated sheep antirabbit immunoglobulin G (IgG) for anti-ACE2 (1:5000, GE Healthcare, catalog number NA934) and HRP-conjugated sheep antimouse IgG for anti-GAPDH (1:10,000, GE Healthcare, catalog number NA931VS)] were incubated at room temperature for 1 h. To facilitate detection of protein bands, we incubated with Immobilon™ Western Chemiluminescent HRP Substrate (Millipore) at room temperature for 5 min. Protein bands were developed onto Super XR-N X-ray films (Fujifilm), scanned by ChemiDoc™ MP Imaging System (Bio-Rad), and densitometry was performed using ImageJ.

### Statistical Analysis

All experiments were conducted and analyzed by investigators that were blinded to the clinical data. Categorial and continuous clinical variables are reported as total number (with relative proportion) and mean ± standard deviation (SD), respectively. Experimental data are presented as means ± standard error of the mean (SEM). The average was calculated when more than one measurement of the same kind of experiment could be acquired in the same patient to obtain one single value for each patient. Normal distribution was tested using Shapiro–Wilk normality test in GraphPad Prism 9. If a variable was normally distributed, significance was tested by a parametric test. Otherwise, the appropriate non-parametric test was used. For the comparison of two groups, we used Student's t and Mann–Whitney tests as parametric and non-parametric test, respectively. One-way analysis of variance (ANOVA) with Holm–Sidak's *post hoc* correction was applied for the comparison of more than two groups that were normally distributed. Kruskal–Wallis test with Dunn's *post hoc* correction was used for the comparison of more than two groups that were not normally distributed. Chi-square test was used for the comparison of categorial data.

Univariate linear regression analyses were performed using IBM SPSS Statistics 25 to test for the following potential confounders and effect modifiers: age, body mass index, arterial hypertension, existing HFrEF, existing atrial fibrillation, history of stroke, diabetes mellitus, and glomerular filtration rate. All variables with *P* < 0.3 in the univariate regression analyses were included in a multivariate model.

To test whether a correlation was significantly different in women and men, the interaction term was calculated by multiplying the independent variable with either 0 or 1 for men and women, respectively. Then, multivariate regression analysis was performed incorporating the independent variable, gender, and the interaction term. If the interaction term's *P* < 0.05, the correlation between the independent variable and the response variable was considered to be significantly different in women and men. For each regression analysis, the regression coefficient B with the 95% confidence interval for each variable was presented. *P*-values below 0.05 were considered as statistically significant.

## Results

### Baseline Characteristics of Study Population

Three hundred twenty-seven patients undergoing elective CABG were included in this study. Polygraphy revealed the presence of moderate to severe SDB (AHI ≥15/h) in 161 patients (49%, Table 1 and Figure 1). In accordance with previous data, patients with SDB were older (*P* = 0.046), had a higher body mass index (*P* = 0.006), and a tendency toward increased prevalence of arterial hypertension (*P* = 0.053) and atrial fibrillation (*P* = 0.080). Accordingly, history of stroke was significantly more common in patients with SDB (*P* = 0.003). Importantly, patients with SDB were more likely to present with HF (81/161 vs. 39/166, *P* < 0.001). In accordance, New York Heart Association (NYHA) functional class and levels of NT-pro BNP (*P* < 0.001) were also significantly increased. Patients with SDB exhibited a significantly reduced LVEF (*P* < 0.001) and an impaired renal function (glomerular filtration rate, *P* = 0.025). By definition, patients with SDB showed a significantly increased oxygen desaturation index (/h, 28.2 ± 15.5 vs. 6.6 ± 4.3*, P* < 0.001) and decreased minimum oxygen saturation (O_2min_ in %, 78.3 ± 7.0 vs. 82.5 ± 6.0, *P* < 0.001), indicating intermittent hypoxemia (Table 2). In patients with SDB, central apnea index (12.5 ± 13.4, *P* < 0.001 vs. no SDB) was higher than the obstructive apnea index (9.7 ± 11.0, *P* < 0.001 vs. no SDB), without statistical difference between the frequency of the abnormal breathing types (*P* = 0.398). Female patients with SDB were significantly older (*P* = 0.022) with more severe HF symptoms (NYHA functional class) (Table 3) than male patients with SDB. On the other hand, there was no sex-dependent age difference for the total cohort (female vs. male, 68.1 ± 9.2 vs. 67.2 ± 8.2 years, *N* = 51 vs. 276, *P* = 0.448, Student's *t*-test). In patients with HF, those with HFpEF exhibited increased obstructive (6.8 ± 8.0 vs. 5.5 ± 8.9, *P* = 0.044) and central apnea indices (10.1 ± 14.3 vs. 6.2 ± 9.6, *P* = 0.083) compared to patients without HFpEF. However, obstructive and central apnea indices were not significantly different, neither in patients without HFpEF (*P* = 0.245) nor in those with HFpEF (*P* = 0.864).

**Table 1:** Baseline characteristics.

	**Total cohort (*N* = 327)**	**No SDB (*N* = 166)**	**SDB (*N* = 161)**	***P*-value**
Age, years, mean ± SD	67.3 ± 8.3	66.4 ± 9.0	68.3 ± 7.5	**0.046**^T^
Male gender, N (%)	276 (84.4%)	138 (83.1%)	138 (85.7%)	0.520^Chi^
Body mass index, kg/m^2^, mean ± SD	28.7 ± 4.4	28.0 ± 4.0	29.4 ± 4.6	**0.006**^T^
Art. Hypertension, N (%)	280 (85.6%)	136 (81.9%)	144 (89.4%)	0.053^Chi^
Diabetes mellitus, N (%)	110 (33.6%)	52 (31.3%)	58 (36.0%)	0.369^Chi^
Hyperlipidemia, N (%)	215 (65.7%)	103 (62.0%)	112 (69.6%)	0.152^Chi^
Atrial fibrillation, N (%)	55 (16.8%)	22 (13.3%)	33 (20.5%)	0.080^Chi^
History of stroke, N (%)	29 (8.9%)	7 (4.2%)	22 (13.7%)	**0.003**^Chi^
**Heart function**				
HF, N (%)	120 (36.7%)	39 (23.5%)	81 (50.3%)	** <0.001**^Chi^
HFrEF, N (%)	25 (7.6%)	6 (3.6%)	19 (11.8%)	**0.005**^Chi^
HFmrEF, N (%)	22 (6.7%)	5 (3.0%)	17 (10.6%)	**0.006**^Chi^
HFpEF, N (%)	73 (22.3%)	28 (16.9%)	45 (28.0%)	**0.016**^Chi^
**NYHA functional class^§^**				
Class I, N (%)	9 (2.8%)	4 (2.4%)	5 (3.1%)	0.701^Chi^
Class II, N (%)	64 (19.6%)	22 (13.3%)	42 (26.1%)	**0.003**^Chi^
Class III, N (%)	43 (13.1%)	12 (7.2%)	31 (19.3%)	**0.001**^Chi^
Class IV, N (%)	4 (1.2%)	1 (0.6%)	3 (1.9%)	0.300^Chi^
NT-pro BNP, pg/ml, mean ± SD	1389.9 ± 3963.0	1033.7 ± 4135.3	1748.6 ± 3762.1	** <0.001**^MW^
LVEF, %, mean ± SD	56.1 ± 9.7	58.5 ± 7.6	53.6 ± 11.0	** <0.001**^MW^
GFR, ml/min, mean ± SD	73.8 ± 21.3	76.2 ± 21.1	71.2 ± 21.4	**0.025**^MW^
Angiotensin-converting enzyme inhibitor or angiotensin II receptor blocker, N (%)	245 (74.9%)	124 (74.7%)	121 (75.2%)	0.924^Chi^

*Values are either the number of patients (relative percentage in parenthesis) or mean ± SD, as appropriate. Comparisons shown in bold values are statistically significant*.

§ *If HF was diagnosed*.

*Chi, chi-square test; GFR, glomerular filtration rate; HF, heart failure; HFmrEF, heart failure with mid-range ejection fraction; LVEF, left ventricular ejection fraction; MW, Mann–Whitney test; NYHA, New York Heart Association; SD, standard deviation; T, Student's t-test*.

**Table 2:** Polygraphy data.

	**Total cohort (*N* = 327)**	**No SDB (*N* = 166)**	**SDB (*N* = 161)**	***P*-value**
Total recording time, min	473.5 ± 59.9	475.3 ± 60.4	471.5 ± 59.5	0.210^MW^
Apnea-hypopnea index, /h	19.7 ± 16.2	7.8 ± 4.1	32.0 ± 14.7	** <0.001**^MW^
Obstructive apnea index, /h	5.8 ± 8.7	2.1 ± 2.2	9.7 ± 11.0	** <0.001**^MW^
Central apnea index, /h	7.1 ± 10.9 (*P* = 0.250^MW^ vs. obstructive apnea index)	1.9 ± 2.2 (*P* = **0.045**^MW^ vs. obstructive apnea index)	12.5 ± 13.4 (*P* = 0.398^MW^ vs. obstructive apnea index)	** <0.001**^MW^
Oxygen desaturation index, /h	17.2 ± 15.6	6.6 ± 4.3	28.2 ± 15.5	** <0.001**^MW^
Minimum oxygen saturation, %	80.4 ± 6.8	82.5 ± 6.0	78.3 ± 7.0	** <0.001**^MW^
Mean oxygen saturation, %	91.7 ± 2.3	92.1 ± 2.0	91.3 ± 2.4	** <0.001**^MW^
Mean heart rate, /min	72.2 ± 12.3	71.7 ± 12.3	72.6 ± 12.4	0.480^MW^

*All values are presented as mean ± standard deviation, and comparisons shown in bold values are statistically significant. An apnea–hypopnea index ≥15/h defined sleep-disordered breathing (SDB)*.

*MW, Mann–Whitney test; TRT, total recording time*.

**Table 3:** Baseline characteristics of women and men with SDB.

	**Female (*N* = 23)**	**Male (*N* = 138)**	***P*-value**
Age, years, mean ± SD	71.6 ± 8.1	67.7 ± 7.3	**0.022**^T^
Body mass index, kg/m^2^, mean ± SD	29.8 ± 4.6	29.3 ± 4.6	0.637^T^
Art. Hypertension, N (%)	21 (91.3%)	123 (89.1%)	0.753^Chi^
Diabetes mellitus, N (%)	8 (34.8%)	50 (36.2%)	0.893^Chi^
Hyperlipidemia, N (%)	14 (60.9%)	98 (71.0%)	0.328^Chi^
Atrial fibrillation, N (%)	6 (26.1%)	27 (19.6%)	0.473^Chi^
History of stroke, N (%)	4 (17.4%)	18 (13.0%)	0.574^Chi^
**Heart function**			
HF, N (%)	12 (52.2%)	69 (50.0%)	0.847^Chi^
**NYHA functional class^§^**			
Class I, N (%)	0 (0.0%)	5 (3.6%)	0.354^Chi^
Class II, N (%)	4 (17.4%)	38 (27.5%)	0.305^Chi^
Class III, N (%)	8 (34.8%)	23 (16.7%)	**0.041**^Chi^
Class IV, N (%)	0 (0.0%)	3 (2.2%)	0.475^Chi^
Apnea–hypopnea index, /h, mean ± SD	32.8 ± 20.3	31.8 ± 13.6	0.349^MW^
Minimum oxygen saturation, %, mean ± SD	77.5 ± 6.1	78.4 ± 7.1	0.342^MW^
GFR, ml/min, mean ± SD	64.2 ± 23.1	72.4 ± 21.0	0.093^MW^

*Values are either the number of patients (relative percentage in parenthesis) or mean ± SD, as appropriate. Comparisons shown in bold values are statistically significant*.

§ *If heart failure was diagnosed*.

*Chi, chi-square test; GFR, glomerular filtration rate; HF, heart failure; MW, Mann–Whitney test; NYHA, New York Heart Association; SD, standard deviation; T, Student's t-test*.

### Female SDB Patients Exhibit HFpEF More Frequently

**Figure 2:**
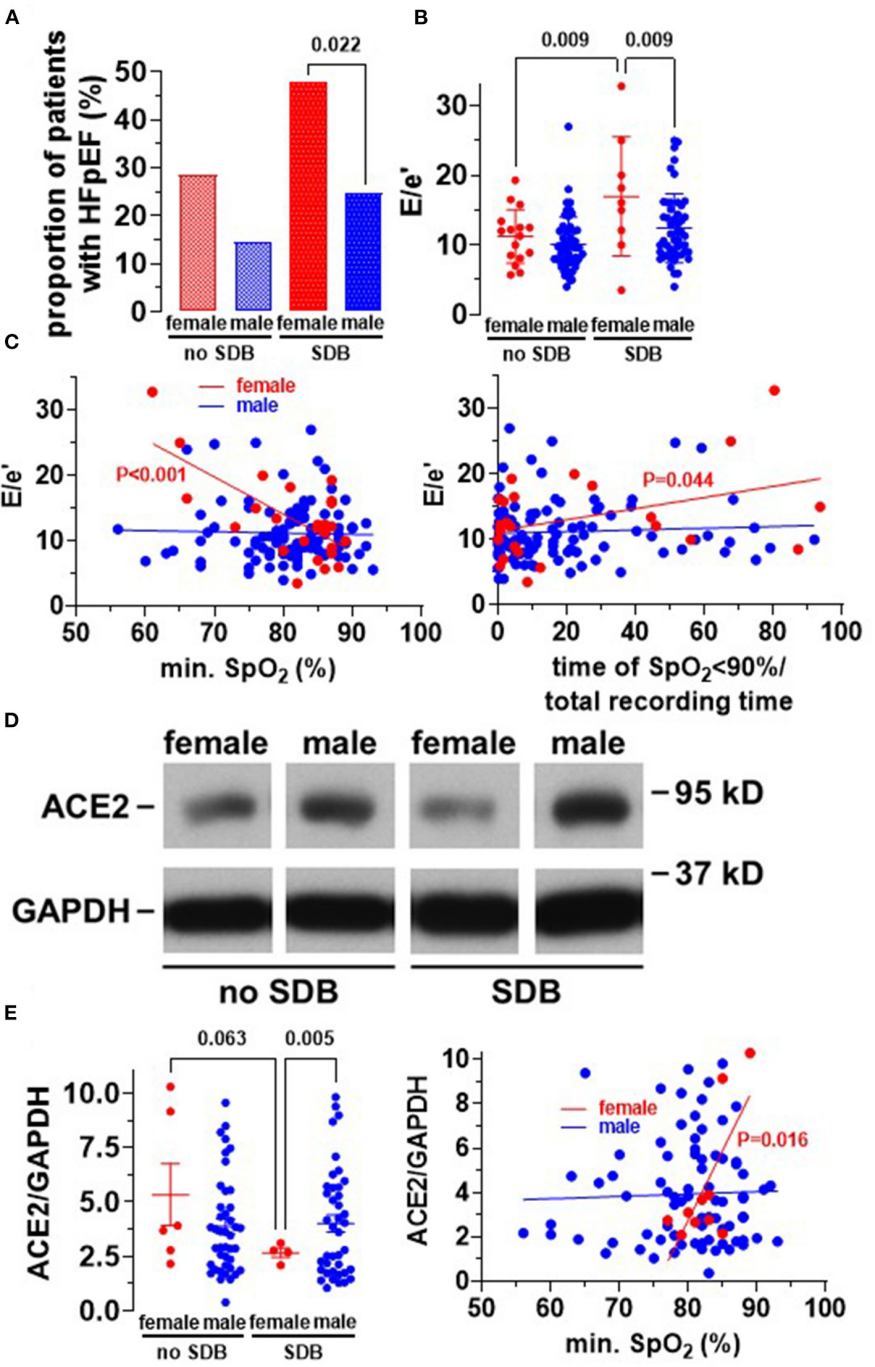
Increased frequency of HFpEF in women with SDB. **(A)** Proportion of patients having heart failure with preserved ejection fraction (HFpEF). Interestingly, HFpEF was significantly more common in women with SDB (*N* = 11/23) compared to men with SDB (*N* = 34/138). **(B)** Accordingly, the severity of diastolic dysfunction, estimated by E/e′, was significantly increased in women with SDB (*N* = 9) compared to women without SDB (*N* = 16) and men with SDB (*N* = 50). **(C)** This resulted in significant correlations between E/e′ with both O_2min_ and time of oxygen saturation <90% (% of total recording time) in women but not in men, indicating that hypoxia may be involved in HFpEF-development in women. **(D)** Original Western blots for the analysis of ACE2 expression in right atrial appendage biopsies. **(E)** Intriguingly, densiometric analyses revealed that ACE2 expression was significantly decreased in women with SDB (*N* = 4), leading to a significant correlation with O_2min_ in women (*N* = 10) but not in men (*N* = 82). Statistical comparisons are based on **(A)** chi-square test **(B,E)**, one-way analysis of variance (ANOVA), and **(C,E)** linear regression analysis.

**Figure 3:**
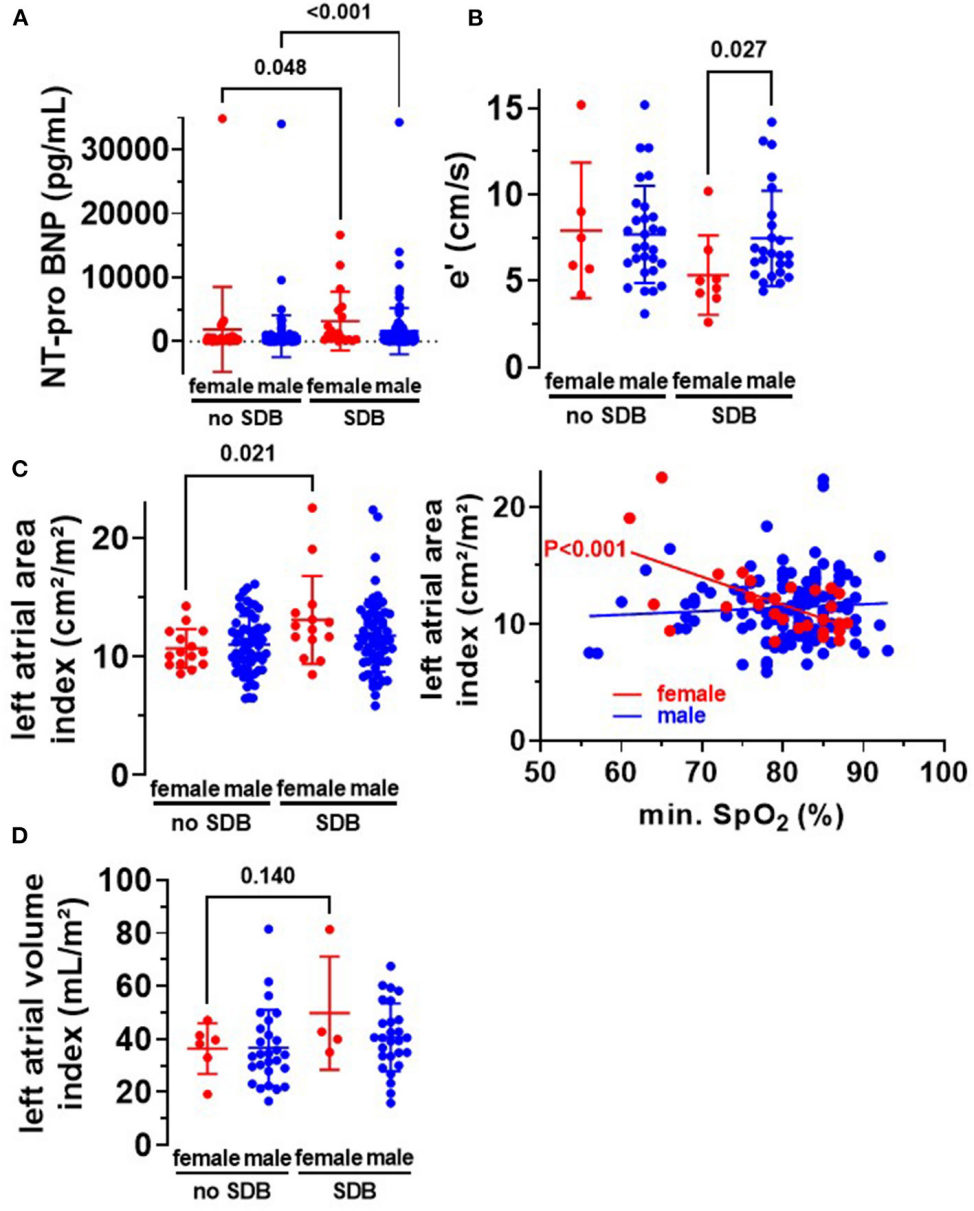
Diastolic dysfunction in women with SDB. **(A)** In accordance with an increased frequency of heart failure in women and men with SDB (Table 1), we observed a significantly increased level of NT-pro BNP in women (*N* = 19 vs. *N* = 27) and men (*N* = 126 vs. *N* = 119) with SDB. **(B)** Early diastolic mitral annulus velocity (e′) was significantly decreased in women with SDB (*N* = 8), indicating diastolic dysfunction. **(C)** Moreover, left atrial area index was significantly increased in women with SDB (*N* = 14) compared to women without SDB (*N* = 15), leading to a significant correlation with O_2min_ in women but not in men. **(D)** Consistently, we observed a trend toward an increased left atrial volume index in women with SDB (*N* = 4 vs. *N* = 6). Statistical comparisons are based on **(A,B)** Kruskal–Wallis test **(C,D)** one-way analysis of variance (ANOVA), and **(C)** linear regression analysis.

As reported in Table 1, HFpEF was significantly more frequent in SDB patients (48/161 vs. 28/166, *P* = 0.016). Intriguingly, this distribution was mainly driven by an increased frequency of HFpEF in female SDB patients (11/23 vs. only 34/138 in male, *P* = 0.022, Figure 2A). Compared to female patients without SDB, women with SDB exhibited significantly increased levels of NT-pro BNP (*P* = 0.048, Figure 3A). In accordance with increased prevalence of HFpEF, there was a significantly increased ratio of transmitral early peak velocity and early diastolic mitral annulus velocity (E/e′) of 17.0 ± 8.5 in women with SDB compared to 11.2 ± 3.9 in women without SDB (*P* = 0.009) and compared to 12.4 ± 5.0 in male SDB patients (P = 0.009, Figure 2B). Consistent with a gender-based difference in diastolic dysfunction in SDB, there was significant negative correlation of E/e′ with O_2min_ only in women (*R*^2^ = 0.470, *P* < 0.001, Figure 2C) but not in men (*R*^2^ < 0.001, *P* = 0.762, Figure 2C). Moreover, this association was significantly different and stronger in women than in men (*P* < 0.001, Table 5). Similarly, E/e′ was also significantly positively correlated with the duration of oxygen saturation below 90% (*R*^2^ = 0.165, *P* =0.044) only in female but not in male patients. Other parameters of diastolic dysfunction were altered in a similar direction: compared to male, female SDB patients exhibited significantly decreased e′ (*P* = 0.027, Figure 3B).

As indicated in Table 1, patients are heterogeneous and present with several comorbidities that could potentially confound our data. Based on these differences, we conducted univariate regression analyses for E/e′ and minimum oxygen saturation (O_2min_), as measure of hypoxia during SDB, age, body mass index, arterial hypertension, existing HFrEF, existing atrial fibrillation, history of stroke, diabetes mellitus, and glomerular filtration rate for all female patients (Table 4). Besides O_2min_, age, glomerular filtration rate, and HFrEF by trend predicted the severity of diastolic dysfunction in women. Female SDB patients were older than female patients without SDB (71.6 ± 8.1 vs. 65.3 ± 9.3 years, *N* = 23 vs. 28, *P* = 0.015). The different age distribution may result in confounding. Therefore, we incorporated age and other important comorbidities into multivariate linear regression analysis. Importantly, after inclusion of all variables with *P* < 0.3 into a multivariate regression model, O_2min_ remained the only independent predictor for the severity of diastolic dysfunction in women (*R*^2^ = 0.534, *P* = 0.042, Table 4).

**Table 4:** O_2min_ independently predicts the severity of diastolic dysfunction in women.

***N* = 25**	**Univariate linear regression analyses**		**Multivariate linear regression analysis Adj**. ***R***^****2****^ **=** **0.534**	
**Variable: E/e^**′**^**	**B (95% CI)**	***P*-value**	**B (95% CI)**	***P*-value**
O_2min_ (%)	−0.569 (−0.830; −0.308)	** <0.001**	−0.333 (−0.652; −0.014)	**0.042**
Age (years)	0.332 (0.082; 0.582)	**0.011**	0.233 (−0.031; 0.497)	0.080
Body mass index (kg/m^2^)	0.346 (−0.206; 0.898)	0.208	0.268 (−0.175; 0.711)	0.219
Arterial hypertension	−3.457 (−10.736; 3.821)	0.336		
Existing HFrEF	12.192 (−0.675; 25.058)	0.062	4.640 (−6.700; 15.980)	0.401
Existing atrial fibrillation	3.606 (−3.659; 10.871)	0.315		
History of stroke	−2.049 (−9.426; 5.328)	0.571		
Diabetes mellitus	2.799 (−2.553; 8.152)	0.291	2.965 (−0.956; 6.886)	0.130
Glomerular filtration rate (ml/min/1.73 m^2^)	−0.107 (−0.197; −0.017)	**0.021**	−0.022 (−0.114; 0.070)	0.616

*Multivariate linear regression analysis revealed minimum oxygen saturation (O_2min_) as an independent predictor for the severity of diastolic dysfunction (i.e., E/e′) in women. P-values shown in bold are statistically significant*.

### Structural Remodeling in Women With SDB

Since HFpEF is also defined by atrial enlargement and hypertrophy, we analyzed these parameters in our patient cohort. Interestingly, in female SDB patients, left atrial volume index was increased by trend (Figure 3D), and left atrial area index was significantly increased compared to women without SDB. The left atrial area index (cm^2^/m^2^) increased from 10.7 ± 1.6 to 13.1 ± 3.7 (*P* = 0.021, Figure 3C). The latter also correlated significantly with O_2min_ in female patients *R*^2^ = 0.363, *P* < 0.001 but not in male patients (Figure 3C). This suggests that hypoxia may be mechanistically linked to HFpEF development and subsequent structural atrial remodeling in women.

**Figure 4:**
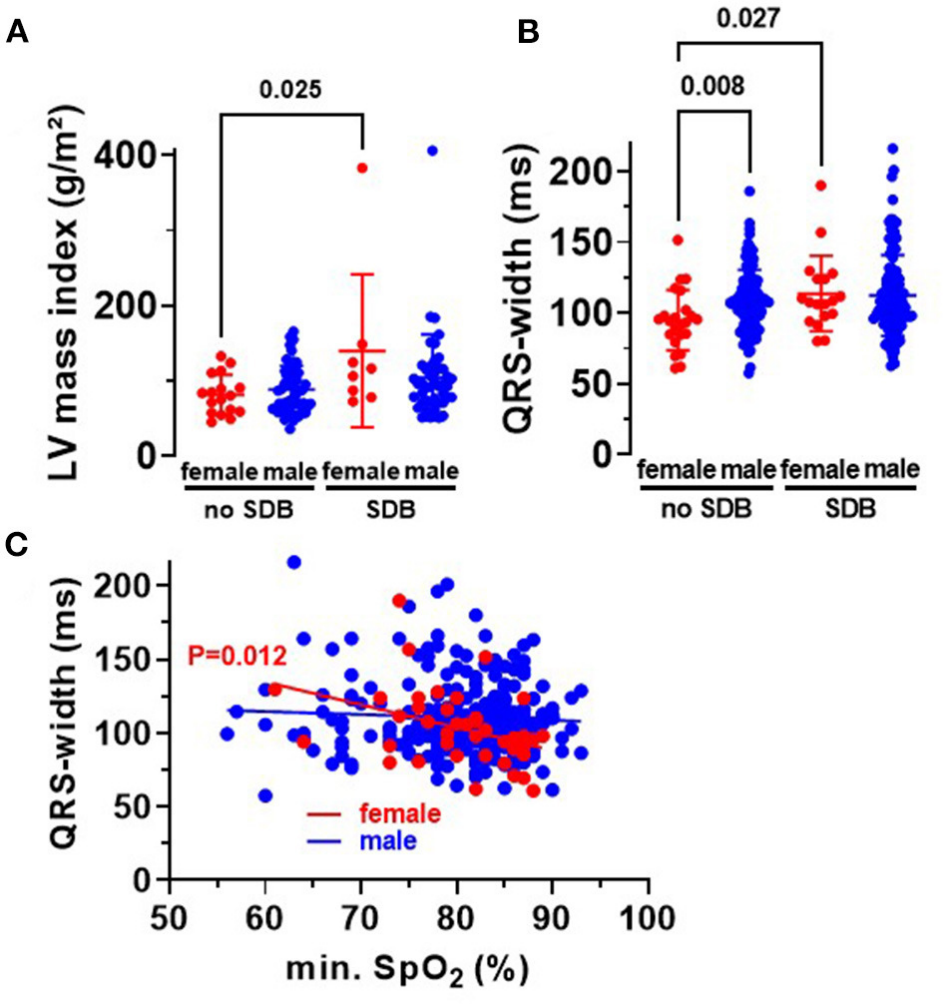
Cardiac remodeling in patients with SDB. **(A)** Left ventricular mass index was significantly increased in women with SDB (*N* = 8) compared to women without SDB (*N* = 17). **(B)** Accordingly, we observed a significantly increased QRS width in women with SDB (*N* = 18) and a significant correlation with O_2min_ in women (*N* = 41) **(C)**, proposing that hypoxemia may be especially relevant for myocardial hypertrophy in women. Statistical comparisons are based on **(A,B)** Kruskal–Wallis test, and **(C)** linear regression analysis.

Beside atrial dilation, left ventricular hypertrophy is also a characteristic for HFpEF. Indeed, we observed a significantly increased left ventricular mass index in women with SDB compared to women without SDB (*P* = 0.025, Figure 4A). Moreover, female SDB patients exhibited a significantly increased QRS width. Compared to female patients without SDB, QRS width (in ms) increased from 94.9 ± 21.3 to 113.7 ± 26.7 in women with SDB (*P* = 0.027, Figure 4B). Consistently, the magnitude of QRS width was significantly negatively correlated with O_2min_ in women (*R*^2^ = 0.152, *P* = 0.012, Figure 4C) but not in men.

Since angiotensin II signaling is a hallmark for HF and myocardial hypertrophy, we measured protein expression of its cleavage enzyme ACE2 in human right atrial appendage biopsies (Western blots, Figure 2D). Intriguingly, for the cohort of patients with SDB, ACE2 expression was significantly decreased in women (to 2.7 ± 0.2) compared to men (4.0 ± 0.4, *P* = 0.005), and there was a strong trend compared to women without SDB (5.3 ± 1.4, *P* = 0.063, Figure 2E). Consistent with a potential mechanistic role of hypoxia, O_2min_ was significantly positively correlated with ACE2 expression but only in women (*R*^2^ = 0.534, *P* = 0.016) and not in men (Figure 2E).

## Discussion

In this study, we characterized HF in high-risk patients with SDB monitoring prior to CABG surgery. While patients with SDB were at increased risk for HF, in general, women with SDB were more likely to present with HFpEF compared to men with SDB. Accordingly, we observed increased cardiac hypertrophy (left ventricular mass index) in women with SDB, possibly due to a decreased myocardial ACE2 expression in female SDB patients.

### Increased Frequency of HFpEF in Women With SDB

Over the last decades, the incidence and prevalence of HF has increased (6, 11). According to the Framingham Heart Study, the overall lifetime risk for developing HF was similar for women and men (16). Despite increasing socioeconomic importance, therapeutic strategies for patients with HFpEF are still limited (6). Most recently, in the PARAGON-HF trial, angiotensin receptor–neprilysin inhibitor sacubitril-valsartan was tested in patients with HFpEF, but treatment failed to reduce the combined endpoint of HF hospitalization and death from cardiovascular causes (17). Therefore, improved understanding of pathomechanisms may favor better characterization and phenotyping of patients with HFpEF, which is essential to find better, personalized HFpEF therapy. Intriguingly, subgroup analyses of PARAGON-HF trial revealed that in patients with HFpEF treatment with sacubitril-valsartan, reduced hospitalization was only observed in women but not in men (18). Indeed, there are remarkable sex differences when considering the entities of HF. Overall, HFpEF has been shown to be more frequent in women than in men (11). However, recent studies have shown that younger HFpEF patients are more likely obese non-white men, while older HFpEF patients are predominantly hypertensive women (19, 20). The different distribution of HF entities across men and women are even more pronounced in older patients (21).

Interestingly, we observed increased frequencies of all HF entities in patients with SDB (Table 1). In addition, LVEF was reduced in patients with SDB, which is in accordance with recently published data (22, 23). While the increased risk for developing HFpEF has been established for female sex and SDB separately (23), currently only sparse data were published reporting sex differences for HFpEF in patients with SDB. SDB is a widespread disease affecting almost one billion people worldwide (1). Interestingly, analyses of 12,608,637 patients from the National Inpatient Sample revealed that 3.8% of patients with obstructive sleep apnea (mean age, 62.5 years) were discharged with HFpEF (23). On the other hand, the prevalence of obstructive sleep apnea was 16.8% in patients with HFpEF and only 5.0% in patients without (23). Importantly, we report here in high-risk patients with severe coronary artery heart disease undergoing CABG a more than 7-fold increased risk for HFpEF with 28% in patients with SDB (Table 1). The risk for HFpEF increases further in women with SDB, showing an HFpEF frequency of 48%. This is in accordance with the study of Hwang et al., where 68% of patients hospitalized due to HFpEF also displayed coronary artery disease, of which 80% underwent revascularization (63% percutaneous intervention and 37% CABG) (24).

### Potential Mechanisms for HFpEF Development in Women With SDB

Until now, several mechanisms have already been proposed to be involved in HFpEF development with secondary atrial remodeling, e.g., ventricular hypertrophy, arrhythmias, fibrosis, and inflammatory processes (10, 25, 26). Here, we show increased left ventricular hypertrophy (Figure 4A) and dilated atria (Figures 3C,D) in women with SDB. Moreover, we observed an increased QRS width in female SDB patients (Figure 4B), a feature consistent with ventricular hypertrophy (27).

Interestingly, angiotensin II signaling has been linked to structural myocardial remodeling (e.g., fibrosis or hypertrophy) and inflammation (12, 28). In contrast, the latter has been shown to be inhibited by ACE2-mediated degradation of angiotensin I to angiotensin (1–9) and angiotensin II to angiotensin (1–7, 12). In accordance with increased myocardial remodeling, we could show that the protective ACE2 expression was decreased in myocardium of women with SDB (Figure 2E). The importance of sex for the regulation of ACE2 expression was confirmed in two independent cohorts of HF patients. Sama et al. found increased plasma levels of ACE2 in male compared to female patients with HF (29).

Several mechanisms of regulation of ACE2 expression have been proposed. Among them, hypoxia, which activates hypoxia-inducible factor 1α (HIF-1α), has already been shown to decrease ACE2 expression possibly by ADAM17-dependent proteolysis and ectodermal shedding of ACE2 (12, 30, 31). On the other hand, our group has recently demonstrated that treatment with either angiotensin-converting enzyme inhibitor or angiotensin II receptor blocker was associated with an increased myocardial ACE2 expression (32). Although 245 (74.9%) of our study patients received either drug, the treatment frequency was similar in patients without (74.7%) and with SDB (75.2%, *P* = 0.924, Table 1), which renders it unlikely to confound our observations.

Interestingly, we show here that myocardial ACE2 expression and severity of diastolic dysfunction (E/e′) correlated with the minimum oxygen saturation O_2min_ only in women but not in men. Moreover, the association between O_2min_ and the severity of diastolic dysfunction was significantly stronger in women compared to men (Table 5). Importantly, after inclusion of all potential confounders in a multivariate regression model, O_2min_ remained the only independent predictor for diastolic dysfunction in women.

**Table 5:** The association of O_2min_ and the severity of diastolic dysfunction is different in women and men.

***N* = 131**	**Univariate linear regression analyses**		**Multivariate linear regression analysis Adj**. ***R***^****2****^ **=** **0.152**	
**Variable: E/e^**′**^**	**B (95% CI)**	***P*-value**	**B (95% CI)**	***P*-value**
O_2min_ (%)	−0.133 (−0.253; −0.013)	**0.030**	−0.019 (−0.146; 0.107)	0.764
Gender	2.112 (−0.070; 4.294)	0.058	46.905 (24.755; 69.056)	** <0.001**
Interaction term	0.020 (−0.007; 0.046)	0.151	−0.550 (−0.822; −0.279)	** <0.001**

*The interaction term was calculated by multiplying minimum oxygen saturation (O_2min_) with either 1 or 0 for women and men, respectively. Multivariate linear regression analysis revealed a significant correlation between the interaction term and E/e′, indicating that the association of O_2min_ and the severity of diastolic dysfunction (i.e., E/e′) was significantly different in women and men. P-values shown in bold are statistically significant*.

In accordance with our finding, expression of HIF-1α has been reported to be increased in female but not in male rat hearts following coronary ligation. This sex difference was observed despite a comparable amount of hypoxia, suggesting that the hypoxia-dependent increase in HIF-1α expression may be larger in female compared to male myocardium (33). In fact, HIF-1α gene has been shown to bear an estrogen response element, and HIF-1α expression was increased by estrogen receptor-α signaling (34).

In contrast, testosterone has been suggested to alleviate cardiac fibrosis by increased ACE2 activity (35–37). Postmenopausal women exhibit testosterone levels orders of magnitude smaller compared to men of comparable age (38). The role of testosterone for the regulation of ACE2 expression was corroborated by a study of (Dalpiaz et al.). They reported a significantly increased myocardial ACE2 activity in male compared to female spontaneously hypertensive rats, which was markedly decreased after gonadectomy in males (37).

Thus, increased estrogen but decreased testosterone levels may predispose women for decreases ACE2 expression in SDB.

### Limitations

Since patients with coronary heart disease and HF are typically more frequently male than female (11), the lower frequency of female patients cannot be avoided in our study design. We have screened every patient, who was scheduled for elective CABG, for eligibility resulting in 15.6% female patients in this study. For such a cohort, a similar fraction of female patients has been reported before (39). Due to variability of anatomy and surgical techniques, right atrial appendage biopsies are often too small to perform experimental analyses. Therefore, we were only able to analyze the right atrium of 10 female (4 with SDB) and 82 male patients (40 with SDB). This represents a fraction of 92/327 patients (28.1%), which is in accordance with previously published studies using this protocol (8). It should be noted that, for this technically demanding analysis, a number of four per group can be frequently found in experimental Western blot studies. Nevertheless, we cannot exclude error in the statistical comparison due to less robustness against outliers, and future studies are needed to confirm our observations.

## Conclusions

In this study, we found a markedly increased frequency of HFpEF in women compared to men with SDB in a high-risk cohort of patients undergoing CABG. Women are often underrepresented in current HF guidelines, and recent trials with HFpEF patients were negative but suggest a potential sex difference. Thus, a better understanding of gender-dependent mechanisms of diastolic dysfunction is urgently warranted. We suggest that there may be a gender difference with respect to myocardial ACE2 expression in response to SDB-dependent hypoxia, favoring hypertrophy and subsequent HFpEF development in women. However, future studies are needed to further delineate these pathomechanisms in detail. Indeed, there are already similar large trials about this topic ongoing, like SDB-CABG-BO1 (DRKS00014665) (40).

## Data Availability Statement

The original contributions presented in the study are included in the article/supplementary material, further inquiries can be directed to the corresponding author/s.

## Ethics Statement

The studies involving human participants were reviewed and approved by University of Regensburg, Bavaria, Germany. The patients/participants provided their written informed consent to participate in this study.

## Author Contributions

SL, PH, MT, LR, CS, LM, MA, and SW contributed to study design. SL, PH, and MT contributed to experimental work. SL, PH, MT, LM, MA, and SW contributed to data analyses. SL contributed to manuscript drafting. PH, MT, LR, CS, LM, MA, and SW contributed to manuscript review and editing. All authors read and approved the final manuscript.

## Conflict of Interest

MA received consulting fees from ResMed, Philips Respironics, Boehringer-Ingelheim, NRI, Novartis, Bayer, and Bresotec, and grant supports from ResMed as well as ResMed Foundation, all outside the submitted work. The remaining authors declare that the research was conducted in the absence of any commercial or financial relationships that could be construed as a potential conflict of interest.

## Abbreviations

ACE2: angiotensin-converting enzyme 2
AHI: apnea–hypopnea index
CABG: coronary artery bypass grafting
HF: heart failure
HIF-1α: hypoxia-inducible factor 1α
HFpEF: heart failure with preserved ejection fraction
HFrEF: heart failure with reduced ejection fraction
LVEF: left ventricular ejection fraction
NT-pro BNP: N-terminal-pro brain natriuretic peptide
O_2min_: minimum oxygen saturation
SDB: sleep-disordered breathing.
